# The Impact of a Neurocollaborative Theraplay® Informed Intervention on the Presentations of Developmental Trauma and Attachment Difficulties in Adopted Children with Prenatal Alcohol Exposure: An Extended Case Study

**DOI:** 10.1007/s40653-025-00715-z

**Published:** 2025-05-31

**Authors:** Jack Purrington, Alan D. Price, Chloe Godfrey, Jacqueline Lynch, Penny A. Cook, Raja A. S. Mukherjee

**Affiliations:** 1 Chrysalis Associates, York, UK; 2 University of Salford, Greater Manchester, Salford, UK; 3 Chrysalis Associates, Grantham, UK; 4 Chrysalis Associates, Sheffield, UK; 5 Surrey and Borders Partnership NHS Foundation Trust, Leatherhead, UK

**Keywords:** Prenatal alcohol exposure, Trauma, Attachment disruption, Adoption, FASD

## Abstract

The UK incident rate of Fetal Alcohol Spectrum Disorders (FASD) in looked after populations is estimated to be 27%. The vast majority of these children enter care due to experiences synonymous with the prodromal stages of developmental trauma and all of them experience some form of attachment disruption. The intersection between developmental trauma, attachment disruptions, prenatal alcohol exposure (PAE), and FASD is an emerging and complex research area however there are currently no evidence based interventions specifically designed for young people with both FASD and early life trauma. Therefore, the purpose of this extended case study is to explore the impact of a novel, practice-based, neurocollaborative Theraplay®-informed intervention on the presentations of developmental trauma and attachment difficulties in adopted children with PAE. The study was based in a specialist trauma and attachment service based in the North of England. A total 16 families engaged in a therapeutic intervention based on the principles of Theraplay, sensory interventions, and Dyadic Developmental Parenting. Outcomes were measured on the Trauma Symptom Checklist for Young Children (TSCYC), the Child Behaviour Checklist (CBCL), the Behaviour Rating Inventory of Executive Function, and the Assessment Checklist for Children. The TSCYC Depression subscale demonstrated a significant difference following therapy with a further three TSCYC scales approaching significance. Clinically meaningful change was also demonstrated for several participants across the outcome measures. Implications and limitations are discussed. It is hoped that this study can provide proof of concept for interventions targeting pre- and post-natal adversities such as PAE and developmental trauma.

**Supplementary Information:**

The online version contains supplementary material available at 10.1007/s40653-025-00715-z.

The term Fetal Alcohol Spectrum Disorder (FASD) is utilised to describe the wide range of lifelong neurobiological differences which are experienced by certain individuals who experience prenatal alcohol exposure (PAE) during gestation (Popova et al., 2023). Worldwide, an estimated prevalence of PAE places Ireland (60.4%), Belarus (46.6%), UK (41.3%), Denmark (45.8%), and Russia (36.5%) as the top five countries with the highest rates of prenatal alcohol exposure; see Popova et al. (2017) for a comprehensive overview by country and region. However, it is important to note that rates of PAE do not coherently correspond to prevalence of FASD in the general population. Research suggests that factors such as frequency, intensity, duration, onset, and pattern of alcohol exposure, alongside genetic and environmental factors may influence the relationship between PAE and FASD (Popova et al., 2023). Separately therefore, Lange et al. (2017) completed a meta-analytic global prevalence study of FASD in children, reporting estimated rates to be 4.8% in Ireland, 3.7% in Belarus, 3.2% in the UK, 3.6% in Denmark, and 2.9% in Russia. However, some of the most prevalent figures included rates of 11.1% in South Africa, 5.3% in Croatia, and 4.5% in Italy; see Lange et al. (2017) for a comprehensive global overview. Active Case Ascertainment, a method which involves actively looking for and diagnosing new cases, is considered the gold standard for assessing the prevalence of congenital conditions such as FASD (Honein & Paulozzi, 1999; Roozen et al., 2016). The only active case ascertainment study of FASD prevalence in the UK was conducted in the Greater Manchester area. McCarthy et al (2021) identified four cases of FASD and a further four cases of probable FASD in a population sample of 220 mainstream schoolchildren aged 8–9 years, giving a prevalence rate between 1.8% and 3.6%. Although this was a small study only local to Greater Manchester, the prevalence estimate is close to that of the international modelling study. However, a larger, national active case ascertainment study for the UK would provide a more reliable estimate. A UK population screening study, which used data from a large longitudinal cohort in South-West England, found that between 6 and 17% of children screened positive for FASD, depending on how missing data were dealt with (McQuire et al., 2019). There is an ongoing effort to establish a national linked longitudinal research database for FASD in the UK, which could improve recognition, understanding and services for people with FASD in the UK (Harding et al., 2024).

Individuals who have experienced PAE with either suspected or confirmed FASD may present with a wide range of behavioural (Fryer et al., 2007; O’Leary et al., 2010), cognitive (Astley et al., 2009; Flak et al., 2014; Li et al., 2008), and neurobiological differences (Donald et al., 2015; Lebel et al., 2011; Sowell et al., 2008). Such individuals will qualify for a diagnosis of FASD under the Scottish Intercollegiate Guidelines Network 156 if there is evidence of pervasive and long-standing brain dysfunction in 3 or more of the following areas based on a neurodevelopmental assessment: motor skills, neuroanatomy or neurophysiology, cognition, language, academic achievement, memory, attention, executive function including impulse control and hyperactivity, affect regulation, and adaptive behaviour including social skills or social communication and if other aetiological factors (genetics and associated comorbidities) can be ruled out as the cause of these difficulties (National Institute of Health and Care Excellence [NICE], 2022).

Reports from Lange et al. (2013) and Gregory et al. (2015) indicate that rates of FASD in looked after children are as high as 52% in Sweden, 40% in Russia, 34% in the USA, 30% in Canada, 28% in Brazil, and 27% in the UK. Most looked after children (> 86% in England for example [Department for Education, 2023]), enter the care system due to experiences synonymous with the prodromal stages of developmental trauma. Notably, children who have experienced early life developmental trauma and subsequent attachment disruptions may also present with a wide range of behavioural (Dubowitz et al., 2002; Milot et al., 2010) and neurobiological differences (Chugani et al., 2001; De Bellis, 2005; Teicher et al., 2006), akin to FASD presentations.

The impact of the intersectionality between PAE, FASD, and early life traumatic experiences is an emerging and complex research area. A small number of published studies have consistently shown a high level of comorbidity between FASD and early life trauma in clinical samples (e.g., Flannigan et al., 2021; Price et al., 2017; Tan et al., 2022). In terms of the combined impact of PAE and early life trauma, studies have tended to show that PAE is a strong predictor of many developmental outcomes, whilst early life trauma is associated with an additional, but weaker, impact on behavioural and mental health outcomes (Astley-Hemingway et al., 2020; Price et al., 2017). Neglect, as opposed to abuse, has also been shown to be related to some additional cognitive difficulties (Rockhold et al., 2023). However, the existing research in this area is limited and does not always acknowledge the relative impact of different forms and varying severity of early life trauma and other pre and postnatal exposures (PPNA). There are no evidence-based interventions specifically designed for young people with both FASD and early life trauma. Given the fact that emerging research suggests that PAE may often be the primary driver behind many of the developmental difficulties seen in young people with both PAE and early life trauma, it is likely that any service or intervention that fails to acknowledge FASD in this population will be less effective than those that do.

There remains a lack of understanding regarding the impact of attachment disruption for care experienced children who have experienced early life developmental trauma experiences and PAE leading to FASD. Studies on the attachment relationships of children with FASD have sometimes shown that insecure or disorganised attachment styles are related to heavier PAE (O’Connor et al., 1987, 1992, 2002) and that there is a potential impact of PAE on the attachment relationship via the mechanism of social difficulties in the infant (O’Connor et al., 1992), but studies in this area have also failed to delineate the relative impacts of PAE and early life trauma. Research exploring the theory of attachment consistently highlights the important and influential nature of the child-caregiver relationship (Ainsworth et al., 1978; Bowlby, 1969, 1973, 1980, 1991; Main, 1995, 1999). The attachment theory highlights that to develop as an adaptive and healthy individual the child is wholly reliant on the development of an emotional bond with their caregiver (Bowlby, 1988). If the caregiver communicates safety and trust in their relational processes in a predictable and consistent manner, the child will develop an adaptive internal working model in which their caregiver is both a secure base and a safe haven for them to survive and thrive in the world (Cooper et al., 2007).

Recent progressions in the field of developmental psychology have built on the key tenets of attachment theory highlighting the protective and restorative nature of parent–child intersubjectivity (see Ammaniti & Trentini, 2009; Cortina & Liotti, 2010; Trevarthen & Aitken, 2001), co-regulation (see Coan et al., 2006; Fosha, 2001; Schore, 1996), and attunement (see Stern, 1985; Legerstee et al., 2007; van Bakel & Riksen-Walraven, 2008). It can be hypothesised therefore, that interventions for care-experienced children who have experienced attachment disruptions, PAE resulting in FASD-like symptomatology, and early life trauma will benefit from interventions which prioritise the attachment relationship alongside being centred around the impact of FASD.

There are several well-established psychotherapeutic attachment-based interventions for care experienced children (see reviews by Bergstrom et al., 2019; Ní Chobhthaigh & Duffy, 2019; Purrington et al., 2023; Schoemaker et al., 2020). A recent trend in interventions designed for adoptive families with adopted children who have experienced both developmental trauma and attachment disruptions have found value in the use of integrative complementary therapies which target multiple focuses concurrently, as opposed to individually and sequentially (see Hunsley et al., 2021; McCullough & Mathura, 2019; Purrington et al., 2022; Ulasińska et al., 2020). These integrative and complementary therapeutic approaches have demonstrated value in: reducing trauma related anxiety, depression, and aggression, reducing peer problems and relational frustration, and improving the quality of attachment, parental confidence, and communication (Hunsley et al., 2021); reducing behavioural regulation and global executive functioning difficulties, reducing self-esteem difficulties, and improving parent–child and child-peer relationships (MuCullough & Mathura, 2019); reducing trauma related anxiety, anger, post-traumatic stress arousal, and sexual concerns, reducing social and thought problems, reducing executive functioning difficulties relating to inhibition, self-monitoring, emotional control, and behavioural regulation, and reducing non-reciprocal attachment behaviours (Purrington et al., 2022); and in reducing parental experiences of anxiety and supporting parents to develop more realistic perceptions of their children (Ulasińska et al., 2020).

There is a relatively small but growing literature on interventions for FASD. Published interventions often focus on improving cognitive, emotional and social functioning of children with FASD, and/or supporting their families and caregivers through psychoeducation, advocacy and support (Price et al., 2025). There is limited data from high-quality studies such as randomised controlled trials (RCT), but available evidence is encouraging. Ordenewitz et al. (2021) identified 25 RCT studies, of which 12 were published since 2015. The interventions were mostly based on improving specific faculties such as mathematics, self-regulation, literacy, attention, and adaptive functioning. Some were based on training and support, while some investigated the potential for choline supplementation. Not all of these were found to be effective but many tended to lead to improvements, although not to the point where children with FASD reached the same level of functioning as typically developing children.

There is considerable overlap in the principles of promising interventions identified in the lived experience model of intervention strategies for the prevention of secondary conditions in individuals with FASD presented by Petrenko et al. (2014) and in the trauma and attachment-based interventions examined in Purrington et al. (2023). Both papers highlight the importance of providing individual support for parents, alongside individual support for children. Whereas the FASD model emphasises the importance of advocacy across the system such as within school settings, and the developmental trauma and attachment disruption interventions emphasise the importance of dyad-based therapeutic work.

In the literature outlining attachment-based interventions above Theraplay® emerges as a prominent and promising approach for adopted children who have experienced attachment disruptions. Theraplay can be described as an activity-based, predominantly non-verbal therapy that utilises “attachment-based play to create better relationships between parents and their children” (Booth & Koller, 1998: p. 308). Intervening in such a way has been found to improve affect regulation (Lindaman & Lender, 2009), trust (Wettig et al., 2011), and behavioural functioning in children (Francis et al., 2017; Weir et al., 2013), alongside promoting sociality and communication (Francis et al., 2017), and reducing trauma symptomology and externalising difficulties (Purrington et al., 2022). Theraplay has been found to have promise for neurodiverse populations including Autistic children (Chang et al., 2021; Coleman & Hong, 2023; Hiles Howard et al., 2018; Simeone-Russell, 2011), children with Attention Deficit Hyperactivity Disorder (Askari et al., 2014; Siu, 2017), and children with intellectual disabilities (Hofstra et al., 2023; Mohamed & Mkabile, 2015; Siu, 2014). Additionally, Theraplay and Theraplay informed practice has been utilised in similar integrative and neurocollaborative formats with adoptive families to positive effect (see McCullough et al., 2016; McCullough & Mathura, 2019; Purrington et al., 2022; Weir et al., 2013).

There is a lack of research exploring dyad-based psychotherapeutic interventions for care experienced children who have experienced early life developmental trauma, attachment disruptions, and PAE leading to the development of FASD-like symptomology. It is important, therefore, to explore therapeutic provision for this cohort. Subsequently, the aims of this extended case study were to provide a preliminary examination of the impact of one possible therapeutic approach which is a practice-based neurocollaborative, Theraplay and sensory informed intervention on the presentations of developmental trauma, attachment difficulties, and fetal alcohol spectrum disorders in a cohort of care-experienced adopted children with prenatal alcohol exposure. A key focus of this preliminary exploration is to ascertain the utility of further study into dyadic, Theraplay and sensory information interventions alongside parental support for this cohort.

## Method

### Ethical Considerations

This project was classified as a service evaluation and is being written up retrospectively, as such, it did not require ethical approval. However, prior to the pre-therapy assessment all participants were provided with consent forms and consented to the following statement:

“*at times, the data collected from questionnaires may be anonymised and used to evaluate the effectiveness of the work we offer, which may also contribute to published research. On these occasions, no personal information will be used, and only numerical data provided from the questionnaires will be included. This data will be collated with other anonymised data collected within the service. Your consent is optional and not consenting to the use of your child’s questionnaire data in this way will not affect your clinical treatment.”*

Finally, all procedures contributing to this work complied with the American Psychological Association ethical standards in the treatment of participants, the British Psychological Society code of ethics and conduct, and the Health and Care Professionals Council standards of conduct performance and ethics. The data analysis was given a favourable opinion by the University of Salford Health Research Ethics Committee in October 2024 (REF: 2024–3171-2298).

### Service & Service Users

This study makes use of clinical records from Chrysalis Associates in the UK. Chrysalis Associates is a specialist trauma and attachment service supporting adoptive families with therapy clinics in four counties in the North of England with a combined population of over 5,000,000. The service consists of three clinical psychologists, six assistant psychologists, and 14 trauma and attachment therapists with specialist training in social work, psychotherapy, art therapy, drama therapy, and occupational therapy. The service is commissioned through local authorities, and referrals are made by each family’s social worker. The service users included within this study were all funded via applications made to the adoption support fund between 2017 and 2018.

Between January 2017 and December 2018, 16 eligible adoptive families received a therapeutic package. The eligibility criteria for this study included: all children must be legally adopted or on an adoption order, had experienced PAE and early life developmental trauma measured through a clinical interview with adoptive parents, and all parent–child dyads had to be displaying signs of attachment difficulties measured through the Marshack Interaction Method assessment (Hitchcock et al., 2008). Adoptive parents completed assessment measures prior to and following the therapeutic intervention and all families completed a neurocollaboarative therapeutic package.

The neurocollaborative therapeutic packages included dyadic family therapy sessions which were predominantly informed by the principles of Theraplay, sensory and mindfulness groups for children only, and therapeutic parenting sessions informed by the principles of dyadic developmental parenting for parents only. Each aspect of the model is described in further detail below.

The sample of children attending therapy was 88% White British, 58% Male, and the average age at pre-therapy was 9.43, ranging from 5 to 14 years of age (*N* = 16, M = 9.437, SD = 2.502), see Table 1 for wider demographic information. The most common therapeutic package included 30 h of therapy divided evenly between therapeutic parenting, sensory group, and dyadic family therapy sessions (M = 58, R = 28–108). Seven families received Theraplay during their dyadic family therapy sessions, five received Theraplay integrated with principles from Dyadic Developmental Psychotherapy (DDP) see Hughes et al. (2015), and Eye Movement Desensitisation and Reprocessing (EMDR) see Gomez and Jernberg (2013), and four received Theraplay integrated with principles from DDP. Finally, the vast majority of families attended 100% of their therapy sessions (M = 95%, R = 77%−100%).

**Table 1:** Participant demographic overview

Family No	PAE Confirmed / Suspected	No. of Sessions	Attendance Rates	Therapy Package*	Models Used in DT**
P1	Confirmed	72	83%	24TP; 24SG; 24DT	T-EDi
P2	Confirmed	30	100%	10TP; 10SG; 10DT	Theraplay
P3	Confirmed	30	80%	10TP; 10SG; 10DT	T-EDi
P4	Confirmed	30	100%	10TP; 10SG; 10DT	T-DDP
P5	Confirmed	60	100%	20TP; 20SG; 20DT	T-DDP
P6	Confirmed	72	100%	24TP; 24SG; 24DT	T-EDi
P7	Confirmed	72	100%	24TP; 24SG; 24DT	T-DDP
P8	Confirmed	30	100%	10TP; 10SG; 10DT	Theraplay
P9	Confirmed	108	100%	36TP; 36SG; 36DT	T-EDi
P10	Confirmed	90	100%	30TP; 30SG; 30DT	T-EDi
P11	Confirmed	72	100%	24TP; 24SG; 24DT	T-DDP
P12	Suspected	108	100%	36TP; 36SG; 36DT	Theraplay
P13	Suspected	75	77%	25TP; 25SG; 25DT	Theraplay
P14	Suspected	30	100%	10TP; 10SG; 10DT	Theraplay
P15	Suspected	28	100%	8TP; 8SG; 12DT	Theraplay
P16	Suspected	30	93%	10TP; 10SG; 10DT	Theraplay

*DT Dyadic Family Therapy, *SG* Sensory Group, *TP* Therapeutic Parenting

*T-EDi Theraplay with EMDR and DDP integrated, *T-DDP* Theraplay with DDP integrated

N.B. The term Theraplay is being utilised to refer to Theraplay-Informed Practice within this table

### Data Collection & Analysis

To evaluate the overall effectiveness of the therapeutic package, a within subject pre-test/post-test design was utilised with adoptive parents from each family completing outcome measures at two time points: pre-therapy (provided no longer than three months prior to the commencement of therapy and returned before commencement) and post-therapy (provided during the penultimate session of therapy and returned within two weeks). The assessment measures included the Trauma Symptoms Checklist for Young Children (TSCYC), the Child Behavior Checklist (CBCL), the Behavior Regulation Inventory for Executive Function parent form (BRIEF), the Behavior Regulation Inventory for Executive Function parent form 2nd edition (BRIEF-2), and the Assessment Checklist for Children (ACC).

Paired samples t-tests with Cohen’s d effect size were used to compare the results of each scale on each assessment measure between pre- and post-therapy, utilising SPSS Version 29.0. Due to the small sample size and multiple tests used here, the primary statistical outcome of interest was effect size rather than statistical significance.

### Assessment Measures

The Trauma Symptom Checklist for Young Children (TSCYC) was administered for children aged 3–12 years. The TSCYC includes 90-items measured across a 4-point Likert scale, providing an evaluation of nine clinical subscales: anxiety, depression, anger, dissociation, sexual concerns, post-traumatic stress (PTS) intrusion, PTS-avoidance, PTS-arousal, and PTS-total (Briere, 2005). The TSCYC attains an overall alpha score of 0.87 and is reported to exhibit suitable convergent validity with the CBCL (Briere et al., 2001; Wherry, et al., 2008). All 16 families returned a fully completed TSCYC at pre-therapy and post-therapy.

The Child Behaviour Checklist (CBCL) was completed for children between 6 and 18 years of age. The CBCL includes 120-items measured across a 3-point Likert scale exploring a range of internalising and externalising difficulties and is reported to have a strong reliability of results, particularly regarding test–retest application with an intraclass correlation of 0.93–1.00 (Achenbach & Rescorla, 2001). Within the sample, 13 families returned a fully completed CBCL at pre-therapy and post-therapy.

The Behaviour Rating Inventory of Executive Functioning 2nd Edition (BRIEF-2) parent form is designed for children aged 5–18 years. The assessment includes 63-items on a 3-point Likert scale which cover thirteen domains of executive functioning (Gioia et al., 2015). Hendrickson and McCrimmon (2019) reported the BRIEF-2 to possess high internal consistency for all index scores in both standardized and clinical samples (coefficients ranging from 0.79 to 0.97). Additionally, a strong test–retest stability was also reported (correlations ranging from 0.67 to 0.92, M = 0.79). Across this sample, 5 families completed the BRIEF and a further 4 families completed the BRIEF-2.

The Assessment Checklist for Children (ACC) is a 120-item psychiatric assessment completed for children aged 5–10 years. The assessment measures behavioural traits, emotional states, and patterns of relating (Tarren-Sweeney, 2007). Tarren-Sweeney (2007) reported the ACC’s total clinical score scale to have a high internal consistency (Cronbach’s alpha = 0.96), and states that the ACC’s validity is supported due to high correlations between the ACC and CBCL total clinical scores (boys: r = 0.89; girls: r = 0.90). Within this sample, 10 families returned a fully completed ACC at both pre-therapy and post-therapy.

### The Neurocollaborative Therapeutic Package

The neurosequential model of therapeutics (NMT), developed by Perry (2009), aimed to introduce a brain-based understanding into psychotherapeutic interventions for children who have experienced early life developmental trauma. The NMT posits that the brain develops from the ‘bottom up’ with the more complex cortical regions developing from the limbic system which itself develops from the diencephalon and brainstem. Subsequently, the NMT outlines that therapeutic work designed for children who have experienced early life trauma should seek to implement interventions in a brain-based sequence, first targeting the brainstem, then the limbic area, and finally cortical brain areas.

In this way Neurocollaborative Therapeutic Practice (NcTP) builds on the principles of Perry’s (2009) NMT and is seen as an advancement utilised to describe the collaborative application of therapeutic interventions which run parallel to one another, targeting two or more brain areas during a psychotherapeutic intervention.

Subsequently, the Neurocollaborative Therapeutic Package examined in this service evaluation included three complementary components: (1) A sensory regulation group intervention for children targeting the brainstem, (2) A course of dyadic therapy for parents and child which utilised principles from Theraplay to target the child’s brainstem and limbic brain areas, when necessary therapists integrated principles from DDP and EMDR to target limbic and cortical brain areas to meet clinical need, and (3) A course of Dyadic Developmental Parenting-informed therapeutic parenting intervention to support carers in taking a neurocollaborative approach with their children.

### Sensory & Mindfulness Group Intervention for Children

The sensory and mindfulness group aspect of the intervention was informed by the Just Right State Program created by Eadaoin Bhreathnach (see Hughes & Bhreathnach, 2018 online pdf pages 18–24). The sensory motor regulation program was designed by an Occupational Therapist and was predominantly delivered by a team of psychologists. This sensory group utilised activities to develop self-regulation, ‘snack and chat’ using regulating food, a body-based attachment and regulation programme, and a story book series (The Scared Gang) to inform children about self-regulation (Bhreathnach, 2008). Age-appropriate mindfulness activities were also completed during these sessions. During these sessions groups of no more than 4 children, aged between 4 and 16, learnt and practised live strategies to help them identify and reflect on their lived experiences.

#### Theraplay-Informed Parent–Child Intervention

The attachment-based play aspect of the intervention was informed by the principles of Theraplay and included varying degrees of structure, engagement, nurture, and challenge activities in response to the needs identified within each individual child-parent dyad (Booth & Jernberg, 2010). These sessions included at least one adoptive parent and the adopted child, and were delivered by trauma and attachment therapists who were trained to Theraplay level 1, level 2, and/or practicum standard, DDP level 1, level 2, and/or practicum standard, and EMDR trained.

At times in which therapists identified clear clinical need to integrate complementary therapeutic approaches to better support families, the models of DDP and EMDR were integrated into the Theraplay-informed sessions. In this instance assimilative integration was adopted (Messer, 1992; Norcross, 2005; Seymour, 2011), in which the intervention remained grounded in Theraplay as a ‘home theory’ with therapists demonstrating a willingness to selectively incorporate practices from DDP and EMDR that aligned with the underlying attachment and regulation-based principles which underpin the model of Theraplay. In the case of the five families who received Theraplay, integrated with principles from DDP and EMDR within this service evaluation; DDP was utilised to help children and their parents co-create shared understanding around the child’s history and give meaning to current interpersonal dynamics being experienced within the family, and EMDR was utilised to support children and parents to install resources and process traumatic experiences.

#### Dyadic Developmental Parenting Informed Therapeutic Parenting Intervention

The therapeutic parenting aspect of the intervention was informed by the principles of Dyadic-Developmental Practice and Parenting and integrated principles from positive behaviour support interventions as appropriate with the family. These sessions were delivered to at least one adoptive parent, by the family’s trauma and attachment therapist.

This weekly hour included psychoeducation around the PACE approach (Playfulness, Acceptance, Curiosity, and Empathy), principles of therapeutic parenting, time reviewing the content of previous dyadic Theraplay-informed sessions to improve reflective capacity, examining and deepening parental understanding of behaviours that challenge and providing positive behavioural support guidance, exploring how the parents can take care of their own emotional wellbeing, and providing psychoeducation on the Theraplay model. This aspect of the therapeutic package followed a weekly workbook designed by the service. Table 2 provides an overview of all components of the therapeutic package.

**Table 2:** Overview of therapeutic package components

Therapeutic components		Purpose and evidence
Attachment and trauma	Theraplay	A recent systematic review (Money et al., 2021) found “promise” for Theraplay, but recommended more research needed. Individual studies have found: improved behavioural regulation and trauma symptoms (Purrington et al., 2022), reduced aggression (Rezaeianzadeh & Yazdanfar, 2024), improved communication and sociality (Francis et al., 2017; Siu, 2014), reduced mother–child anxiety (Smithee et al., 2021), improved behavioural functioning (Francis et al., 2017; Weir et al., 2013), improved interpersonal relationships, reduced interpersonal distress, improved overall mental health functioning (Weir et al., 2020), building of assertiveness, self-confidence, and relational trust (Wettig et al., 2011), positive increases in affect regulation (Lindaman & Lender, 2009), and reduced internalising symptoms (Siu, 2009)
	DDP	Three interventions within Purrington et al., (2023) systematic review reported that DDP helped parents to develop an increased level of understanding of their child’s mind and behaviour and that DDP is a different approach to parenting which can facilitate positive outcomes (Agbayani, 2014; Turner, 2012; Wingfield & Gurney-Smith, 2019)
	EMDR	Several pieces of literature (Gomez & Jernberg, 2013; Ogden & Gomez,^ 2013^; Rodwell & Norris, 2017; Sun-Reid, 2012) each discuss the use of EMDR within Theraplay, however these are more practitioner text books and case studies and less empirical evidence. Main evidence base for EMDR comes from non-looked after populations e.g., NICE guidelines recommends EMDR for children if TF-CBT may not be suitable. For children with experiences of developmental trauma, attachment disruption, and PAE often TF-CBT Is not suitable due to difficulties in regulation
PAE/FASD & Attachment Trauma	Therapeutic Parenting Support	Gosling and Purrington (2023) and Shepherd et al. (2023) both qualitative studies, found themes of importance of therapeutic parenting support for carers of looked after children during covid. Therapeutic parenting is a core part of DDP as well as shown in themes above – it is also part of the Theraplay Model. Parenting support has also been found to be a beneficial for parents of children with FASD see Burkhart et al. (2024)
PAE/FASD	Sensory Group therapy	Sensory processing difficulties are common in children with PAE/FASD, who may exhibit both sensory seeking and sensory avoiding tendencies. It has been recommended that sensory components are included in occupational therapy interventions for children in this population (Fjeldsted & Xue, 2019)

## Results

### Cohort Analysis: Paired t-tests and Effect Size

A series of paired samples t-tests was conducted to calculate the effect sizes of any changes between pre and post therapy on all subscales of the ACC, BRIEF/BRIEF-2, CBCL, and TSCYC. In total, there were 65 subscales across the five measures. First, significance testing was used to identify subscales of particular interest. Bonferroni corrections were conducted within each measure, giving adjusted alpha levels of 0.003 (ACC), 0.005 (BRIEF), 0.004 (BRIEF-2), 0.003 (CBCL), and 0.006 (TSCYC). Only 1 subscale (TSCYC depression) showed a significant difference at the adjusted alpha level. A further four subscales were significant at the unadjusted 0.05 alpha level, three of which were also from the TSCYC measure (see Table 3). Within these results, the effect sizes were large, ranging from 0.58 for Ar-PTS to 0.88 for TSCYC depression. Table 3 shows these results plus the 95% confidence intervals for Cohen’s d in each of these results. All of these tests yielded effect sizes with wide confidence intervals, and only the TSCYC depression subscale has its lower boundary above 0.1.

**Table 3:** Selected results of paired samples t-tests

Measure	Subscale	Sample size	p-value	Cohen’s d	95% CI (Cohen’s)
TSCYC	Depression	14	0.003**	0.88	0.25, 1.50
	Anxiety	14	0.02*	0.65	0.06, 1.20
	Ar-PTS	14	0.03*	0.58	0.00, 1.14
	Tot-PTS	14	0.02*	0.59	0.01, 1.15
CBCL	Tho Pro	13	0.02*	0.64	0.03, 1.25

**significant following Bonferroni correction; *approaching significance

### Individual Analysis: Clinically Meaningful Improvements

Overall, the 16 participants completed a combined total of 48 pre-therapy-post-therapy evaluations and on 30 (62.5%) of these occasions at least one clinically meaningful improvement was reported per outcome measure. The number of each participant’s clinically meaningful improvements across all scales ranged from 0–15 (M = 5.6), with all but one family reporting their child to have experienced at least one clinically meaningful reduction in symptomology across the four assessment measures, as shown in Table 4. The cohort of families can be roughly divided into three main intervention response groups: a high response group which includes P16, P5, P10, P4, P7, P9, which reported clinically meaningful improvements on 15–8 scales (M = 9.7); a moderate response group which includes P6, P1, P2, P12, and P14 which reported clinically meaningful improvements on 6–3 scales (M = 4); and a low response group which includes P8, P11, P13, and P3 which reported clinically meaningful improvements on 1–0 scales (M = 0.75).

**Table 4:** Number of clinically meaningful improvements across measures per participant

Family No	Number of Scales reducing pre-therapy to post-therapy: Clinical to Average, (Clinical to Borderline), & [Borderline to Average]				
	TSCYC: 9 Scales	CBCL: 17 Scales	BRIEF/BRIEF-2: 11/13 Scales	ACC: 15 Scales	Total: 52/54 Scales
P1	2, (1)	0	N/A	(2)	2, (3)
P2	[1]	1	N/A	[1]	1, [2]
P3*	0	0	0	0	0
P4*	7	0	(3)	0	7, (3)
P5	0	3	3, (3), [1]	N/A	6, (3), [1]
P6	4, (1)	0	(1)	N.A	4, (2)
P7	1	1	[6]	N/A	2, [6]
P8	[1]	N/A	N/A	N/A	[1]
P9	1, (1), [1]	5	0	0	6, (1), [1]
P10	2, (1)	6	N/A	N/A	8, (1)
P11	0	N/A	N/A	1	1
P12	0	0	[1]	[2]	[3]
P13	[1]	0	N/A	N/A	[1]
P14	1, (1), [1]	N/A	N/A	0	1, (1), [1]
P15*	[1]	3	(3), [1]	0	3, (3), [2]
P16*	3, (2)	9	0	[1]	12, (2), [1]

*Completed the BRIEF-2 meaning these participants completed 54 total scales

*N/A* Not applicable as this outcome measure was not returned at post-therapy

The difference in the number of clinically meaningful improvements reported between these groups may not necessarily be reflective of the interventions capacity to bring about such changes in certain families. It may rather reflect the interventions capabilities to bring about clinically meaningful changes for families with children who are experiencing a greater severity of pre-therapy difficulties. For example, P3 did not report any clinically meaningful developments following therapy, however P3 also commenced therapy reporting average levels of difficulties on over more than half of all outcome scales assessed (*N* = 28; 51.8%). Whereas P16 reported the greatest number of clinically meaningful developments and commenced therapy reporting average difficulties on less than a third of scales assessed (*N* = 16; 29%).

Moreover, the four outcome measures each display different participants as having the greatest level of clinically meaningful changes at post-therapy. P6 and P16 reported the greatest number of clinically meaningful scale improvements on the TSCYC each reporting 5 scales to have improved by a clinical classification boundary; on the CBCL P16, P10, and P9 reported the greatest number of clinically meaningful scale improvements with 9, 6, and 5 scales respectively; P5 reported the most clinically meaningful improvements on the BRIEF, with 7 scales, and on the BRIEF-2 this was P15 with 4 scales; whereas on the ACC, P1 and P12 each reported two clinically meaningful improvements. Therefore, the intervention approach appears to be able to flexibly meet the unique presenting needs of each family and child and facilitates clinically meaningful therapeutic change in diffing ways for differing families across four broad spectrums of trauma, emotional and behavioural difficulties, executive functioning, and attachment and mental health.

The TSCYC appeared to reflect the greatest number of positive changes with 75% of the families who completed both pre-therapy and post-therapy TSCYC evaluations reporting at least one scale to be evidencing clinically meaningful positive improvements. This was followed by the combined BRIEF/BRIEF-2 with 66%, the CBCL with 53%, and the ACC with 50%. Table 5 details the scales which demonstrated the greatest number of clinically meaningful changes between the pre- and post-therapy assessments.

**Table 5:** Scales reporting the greatest number of clinically meaningful improvements

Name of Scale	Number of Participants Reporting Clinically Meaningful Changes (%)	Clinical to Average	Clinical to Borderline	Borderline to Average
TSCYC Depression	8 (50%)	6	2	-
TSCYC Anxiety	4 (25%)	1	2	1
TSCYC PTS-Intrusion	4 (25%)	3	-	1
TSCYC Dissociation	4 (25%)	3	-	1
TSCYC Anger	4 (25%)	2	-	2
CBCL Externalising	3 (23%)	3	-	-
CBCL Total Problems	3 (23%)	3	-	-
CBCL Thought Problems	3 (23%)	3	-	-
CBCL Aggressive Behaviours	3 (23%)	3	-	-
CBCL Conduct Problems	3 (23%)	3	-	-

-: no changes made

Conversely, there were a small number of scales which did not report any clinically meaningful changes despite the opportunity to do so. These included the CBCL Somatic Complaints, Rule Breaking Behaviour, and Anxiety Problems scales; the BRIEF scale of Shift; and the BRIEF-2 scales of Inhibit, Shift, Initiate, Behaviour Regulation Index, Emotional Regulation Index, Cognitive Regulation Index, and Global Executive Composite, these scores are displayed in supplementary material one, alongside all raw scores.

## Discussion

This extended case study aimed to retrospectively evaluate the impact of a service based, neurocollaborative, Theraplay® informed intervention on the presentations of developmental trauma and attachment difficulties in adopted children with prenatal alcohol exposure. Despite promising results, it is important to note that the small sample size and multiple variables do not produce generalisable conclusions nor provide reliable evidence of efficacy for the intervention. It is also possible that the results are an artifact of the statistical phenomenon of regression to the mean, whereby those with extreme values on a scale are likely to, at second measure, be closer to the mean – giving the impression of improvement. Rather, this case study forms part of a preliminary analysis which is intended to ascertain the utility of further exploration of dyadic, Theraplay and sensory informed interventions with parenting support for care experienced adoptive children with prenatal alcohol exposure. Subsequently, a 4-year retrospective service evaluation of outcomes between 2020 and 2024 is currently being completed evaluating the use of principles of Theraplay, the alert model, and parenting support sessions, with a much greater sample size and comparisons to a non-PAE control group.

### Changes in Trauma Related Presentations

Although intervention efficacy cannot be purported due to limitations in the scientific rigour of the study design, one interesting feature of the dataset were the changes observed in the TSCYC. Seven of the nine TSCYC scales presented with either clinically meaningful changes, statistically significant changes, or trends towards statistical significance with strong effect sizes. The TSCYC Depression scale was the only scale which demonstrated statistically significant changes alongside clinically meaningful reductions in symptomology, and the TSCYC Anxiety scale trended towards statistical significance with clinically meaningful reductions.

These positive post-therapy improvements can be contextualised in current theoretical understanding of the facilitators for trauma processing. Principles of trauma processing which are drawn upon within successful evidence-based treatments for psychological trauma such as trauma focused cognitive behavioural therapy and EMDR often include the establishment of safety/stabilisation, and/or the development of a coherent trauma narrative, and/or the adaptive integration/consolidation of this narrative into the current time, place, and space that the individual is operating within. All 16 families received dyadic family therapy informed by the principles of Theraplay which is understood to enhance relational trust (Wettig et al., 2011), improve affect regulation skills (Lindaman & Lender, 2009), contribute towards improved interpersonal relationships (Weir et al., 2020), and reduce trauma related symptoms (Purrington et al., 2022) and thus is seen to effectively establish safety within the parent–child relationship. It is understandable that experiencing enhanced affect regulation and trust within the current primary attachment relationship would improve trauma related depression symptoms in a child experiencing trauma related depression symptoms resulting from a trauma experienced within a previous primary attachment relationship.

Promising improvements in trauma-related affective scales of depression and anxiety is particularly noteworthy within the context of PAE. Affect regulation difficulties including controlling, modulating, and adaptively expressing emotional responses is particularly common in children with PAE (Cook et al., 2016). PAE can cause permanent structural brain alterations and acts as a stressor which leads to dysregulation of the hypothalamic adrenal pituitary axis (HPA), resulting in increases in HPA tone and/or activity (Díaz-Miranda et al., 2021). Trauma-related anxiety and depression symptoms for children with PAE and post-natal experiences of developmental trauma and attachment disruption can be contextualised using the stress-diathesis model (Ingram & Luxton, 2005) with PAE increasing a child’s inherent vulnerability to affective disorders and post-natal trauma being the environmental activator to cause clinically significant presentations of these symptomologies. Considering the increased risk factors for affect dysregulation within this cohort of children, the positive changes demonstrated within this dataset on the scales of TSCYC anxiety and depression are in equal parts unexpected and significantly promising for the neurocollaborative Theraplay-informed intervention.

### Relational Safety

The principles of Theraplay are seen to be critical for this cohort. There is evidence to suggest that PAE is related to attachment insecurity (O’Connor et al., 1987; 1992; 2002) whilst attachment disruptions following experiences of interpersonal trauma within a primary attachment relationship also contribute to insecure or disorganised attachment profiles. It is unsurprising therefore, that all children within this study reported some form of attachment difficulty. It also possible that the unique intersection between PAE and post-natal attachment injuries may contribute to more complex patterns of relating within primary attachment relationships for children who have experienced both exposures, further highlighting the importance of relational security and the role of the principles of Theraplay within this intervention.

Theraplay was utilised to develop a sense of relational security within the primary attachment relationship and from this position of relational safety four families also received an integrative intervention based on the principles of Theraplay and DDP and a further five families received an integrative intervention based on the principles of Theraplay, DDP, and EMDR. The application of DDP was designed to facilitate a process of collaborative sense-making for these nine families, in which a new and coherent meaning could be provided to traumatic past experiences to help these experiences become conceptualised in the presence of a different, safer experience within the here-and-now. Equally, the integration of EMDR was designed to specifically support children in adaptively processing blocked or unprocessed traumatic memories. It is understandable therefore, that the utilisation of this constellation of therapeutic approaches, with a particular reference to principles of Theraplay, would predominantly impact trauma symptomology at post-therapy.

In line with this, the assessment scales that reported the greatest number of clinically meaningful improvements across participants were mostly externalised behavioural symptoms such as presentations of depression, anxiety, anger, and dissociation on the TSCYC and experiences of externalising, aggressive behaviour, and conduct problems on the CBCL. Again, this can be contextualised in previous literature. Theraplay has been found to improve behavioural functioning (Francis et al., 2017; Purrington et al., 2022; Weir et al., 2013) and reduce aggression (Rezaeianzadeh & Yazdanfar, 2024). It can be hypothesised that experiences of additional safety and depth within the attachment relationship may have contributed to these children being better able to express their needs and seek comfort from their parents as opposed to using their behaviour to communicate unmet or hidden needs.

### Therapeutic Parenting Support

It is possible that the therapeutic parenting support sessions informed by the principles of DDP, and more specifically the model of dyadic developmental parenting, may have contributed to improvements on scales evaluating externalising behaviours. In qualitative evaluations of DDP, themes of parents and carers reporting increased understanding of their child are consistently reported (Agbayani, 2014; Turner, 2012; Wingfield & Gurney-Smith, 2019) and indeed the application of dyadic developmental parenting within an integrative fashion with other therapies has seen positive outcomes on measures of externalising behaviours (see McCullough & Mathura, 2019; Purrington et al., 2022). It may be the case that the process of developing an enhanced understanding of their child’s behaviour may have altered the manner in which parents completed the post-therapy self-report measures. For example, when initially completing the CBCL scale of Conduct Problems, carers may have perceived their child as unwilling to engage in certain behaviours, whereas post-intervention the parents’ understanding of developmental trauma, attachment difficulties, and the impact of PAE may have led to more compassionate responses.

The importance of providing support for parents is emphasised in Petrenko et al. (2014) exploration into promising features of interventions to support the prevention of secondary conditions for children with FASD. Common secondary conditions for children with FASD include conduct disorders and oppositional defiance disorders (Lange et al., 2018). Consequentially, supporting parents with their experiences of the psychosocial and relational impact of parenting a child with conduct and oppositional defiant experiences is likely to have been a particularly impactful aspect of the intervention, potentially contributing to the positive changes seen on the CBCL.

### Intervention Flexibility

It is also worth considering that the families commenced therapy with differing therapeutic goals and needs specific to their child and family. This is reflected in certain families reporting the greatest changes in trauma domains compared to other families reporting the greatest changes in behavioural and emotional domains meanwhile other families reported observing the greatest changes in attachment domains with others reporting these changes in measures of regulation. This can be seen to be an asset of the neurocollaborative approach when working with children who have experienced PPNA. The impact of pre-natal adversity such as PAE on the developing brain can be extensive with brain functions responsible for academic achievement, adaptive behaviour and social skills, attention, cognition, communication, executive function, memory, and motor skills being impacted (Cook et al., 2016). In addition, post-natal adversity such as developmental trauma and attachment disruption can result in children presenting with dysregulated activity within the hypothalamus, corpus callosum, and cerebellum (Blithikioti et al., 2022; Graziano et al., 2021; Raise-Abdullahi et al., 2023), orbiofrontal cortex and anterior cingulate cortex (Almeida et al., 2022; Bounoua et al., 2022; Rong et al., 2023), and amygdala (Nogovitsyn et al., 2022; Oh et al., 2022). Therefore, therapy seeking adoptive families who have children who have experienced PPNA can attend therapy with a wide range of presentations. These presentations range from ‘crash and bump’ children who are sensory seeking, to shutdown children who are sensory avoidant, and children that seek both at varying times; children experiencing huge waves of emotions such as anger, to children overwhelmed by their emotions who disconnect from their feelings; and children with high control needs to those who survive by fawning and seeking others to be in charge. Considering this, it is clear to see every child who has experienced PPNA will require a uniquely tailored therapeutic intervention. The flexibility of neurocollaborative therapeutic practice to integrate a range of intervention approaches that best meet the idiosyncratic therapeutic needs of each individual child and family is a significant strength of the approach for cohorts of PPNA children.

The principles of neurocollaborative therapeutic practice accommodates for the breadth of presentations of children who have experienced PPNA by being responsive to the therapeutic needs within the room. For example, P6 and P16 exhibited the greatest number of clinically meaningful scale improvements on the TSCYC as the impact of these children’s PPNA may have necessitated a greater focus on traumatic experiences whereas P5 and P7 exhibited the greatest number of clinically meaningful scale improvements on the BRIEF as the impact of these children’s PPNA may have been increasingly regulation-based. This suggests that the flexibility of the neurocollaborative Theraplay-informed intervention contributes to the meaningful accommodation of the breadth of presentations resulting from PPNA to the benefit of children and their families.

### Scales Resistant to Change

However, it is important to reflect on the BRIEF-2 which reported the greatest number of scales reporting no clinically meaningful change, despite being eligible for such change. These scales included Inhibit, Shift, Initiate, Behaviour Regulation Index, Emotional Regulation Index, Cognitive Regulation Index, and Global Executive Composite. These findings broadly map onto the current understanding of the BRIEF-2 for children with FASD in the UK (Mohamed et al., 2019). The original version, the BRIEF, also reported no changes on the Shift scale. One possible reason for this is that cognitive difficulties like executive dysfunction are more likely the result of prenatal alcohol exposure than early life trauma. Studies in the emerging field of PAE and trauma have shown that the additional impact of trauma tends to be related to mental health and behavioural differences more so than cognitive differences (Flannigan et al., 2021; Astley-Hemingway et al., 2020; Price et al., 2017), except in the case of severe or long-term neglect (Rockhold et al., 2023). So, a therapeutic package designed primarily for trauma and attachment difficulties may be less likely to lead to improvement in PAE-related difficulties such as executive functioning and more likely to lead to improvement in trauma-related difficulties such as emotional and behavioural presentations. However, this would be neither surprising nor discouraging – the identification of a treatment option that improves any symptoms in children with both PAE and early life trauma would be a novel and valuable finding.

Another interpretation of the finding that this treatment package was not related to improvements in executive functioning is to consider that the sensory and mindfulness children’s group which aimed to improve self-regulation skills was ultimately an ineffective aspect of the intervention. This may have been due to the group delivery format as certain groups included children with ages ranging from 6 to 16 years. This resulted in increased difficulty in cultivating engagement in this aspect of the intervention. Additionally, the unique needs and presentations of each child occasionally produced an environment in which children within the same group would cause each other to experience dysregulation due to the contradicting needs of their individual psychosocial, emotional, and sensory regulatory diets.

### Considerations for Improvements to the Therapeutic Approach

There is a growing evidence base for the use of the sensory program, the alert model (Nash et al., 2015; Soh et al., 2015; Wells et al., 2012) for children with PAE who exhibit FASD symptoms. Therefore, the application of this approach in a 1-to-1 format may be an increasingly beneficial intervention aspect in future. Additionally, a recent Dyląg et al. (2024) review found that children with PAE with fetal alcohol syndrome benefit from engaging in therapeutic activities that facilitate movement of the body, subsequently proposing the integration of physical activity into therapeutic interventions. It is understandable therefore, that the nine families within this study that received principles of DDP, a predominantly stationary talk-based therapy, may have experienced reduced changes. In lieu of the findings from this study, these changes have already been made by the service and the neurocollaborative theraplay-informed intervention now includes 10 sessions of parent–child therapy based on the principles of Theraplay, 10 sessions of sensory therapy based on the principles of the alert program, and 10 sessions of therapeutic parenting support. An additional exploration is currently taking place of this version of the intervention.

### Strengths. Limitations. & Implications for Future Research

This extended case study presents a practice-based sample of therapy-seeking adoptive families with adopted children who have experienced PPNA including PAE, developmental trauma, and attachment disruptions. We believe this is the first study of its kind evaluating a neurocollaborative intervention which seeks to make preliminary steps towards bringing together interventions for both pre-natal and post-natal adversities for children, parents, and the relational dyad into one cohesive therapeutic package.

The study has several limitations including the small sample size and multiple variables meaning that it is not possible to make generalisations. Rather this study should be received as a preliminary analysis which attempted to ascertain the utility of further exploration into neurocollaborative therapeutic practices for therapy seeking families with children who have experienced PPNA. The results may equally be explained due to the regression to the mean effect and therefore the inclusion of a control group for future studies is imperative. It is also impossible to provide inferences on sustainable effects given outcome data was only collated at two time points. It is hoped that this extended case study can be seen as providing proof of concept and can be used as such to warrant further exploration into the integration of Theraplay-informed activities and sensory activities for children who have experienced PAE, developmental trauma, and attachment disruption.

This preliminary analysis indicates that a further study with a greater sample size alongside a non-PAE comparison group is warranted in order to be able to make wider inferences regarding the neurocollaborative Theraplay-informed therapeutic package. This study is currently being completed and evaluates the impact of principles of Theraplay for the parent–child dyad, principles from the alert program for the child delivered in a 1-to-1 format, and principles of parenting support for the parents with this intervention being delivered to adoptive families with PAE and non-PAE children.

## Conclusion

This extended case study evaluated the impact of a neurocollaborative Theraplay® informed intervention on the presentations of developmental trauma and attachment difficulties in adopted children with prenatal alcohol exposure. Due to the small sample size, multiple variables, and absence of a control group no generalisable conclusions can be made. The greatest clinically meaningful improvement was seen on the TSCYC. Within the cohort all but one child reported at least one clinically meaningful improvement across the post-therapy outcome scales and half the cohort reported clinically meaningful improvements on six scales or more. There is an absence of research examining interventions for families with children who have experienced PPNA, with a particular reference to PAE and post-natal developmental trauma and attachment disruptions. This absence of understanding has led caregivers of children with FASD or suspected FASD in the UK to report feeling frustrated at the lack of training and knowledge of FASD among service providers, and the lack of appropriate services for their children (Price, 2019). It is hoped that this preliminary extended case study will encourage further, higher quality research within this field to better position service providers to meet the needs of children who have experienced PPNA, with a particular reference to PAE, developmental trauma, and attachment disruptions.

## Supplementary Information

Below is the link to the electronic supplementary material.

Supplementary file1 (DOCX 19.1 KB)
