# Supplementary material for: The Impact of a Neurocollaborative Theraplay® Informed Intervention on the Presentations of Developmental Trauma and Attachment Difficulties in Adopted Children with Prenatal Alcohol Exposure: An Extended Case Study
Source: J Child Adolesc Trauma. 2025 May 31;18(3):555–71. doi: 10.1007/s40653-025-00715-z (PMC12433427; doi:10.1007/s40653-025-00715-z)
Supplement: Supplementary file 1 — Supplementary file1 (DOCX 19.1 KB) [file 40653_2025_715_MOESM1_ESM.docx]

**Supplementary table:**

**Results of Paired t-tests**

| **Scale** | **Subscale** | ***p*** | ***d*** |
| --- | --- | --- | --- |
| ACC | Sexual Behaviour | 0.43 | -0.05 |
|  | Pseudomature | 0.11 | 0.42 |
|  | Non-Reciprocal | 0.12 | 0.40 |
|  | Indiscriminate | 0.07 | 0.50 |
|  | Insecure | 0.26 | 0.22 |
|  | Anxious/Distrustful | 0.09 | 0.46 |
|  | Abnormal Pain Response | 0.24 | 0.23 |
|  | Food Maintenance | 0.39 | 0.09 |
|  | Self-Injury Index | 0.02 | 0.80 |
|  | Self-Injury Pica Index | 0.12 | -0.40 |
|  | Suicidal Disclosure | 0.29 | 0.19 |
|  | Total Clinical Score | 0.12 | 0.39 |
|  | Negative Self-Image | 0.23 | 0.25 |
|  | Low Confidence | 0.48 | 0.02 |
|  | Total Composite | 0.34 | 0.13 |
| BRIEF | Inhibit | 0.26 | -0.36 |
|  | Self-Monitor | 0.30 | 0.29 |
|  | Shift | 0.10 | -1.07 |
|  | Emotional Control | 0.39 | -0.15 |
|  | Initiate | 0.14 | -0.64 |
|  | Working Memory | 0.42 | 0.11 |
|  | Plan/Organise | 0.18 | 0.53 |
|  | Task-Monitor | 0.20 | 0.50 |
|  | Organisation of Mats | 0.13 | 0.71 |
|  | BRI | 0.44 | -0.09 |
|  | ERI | 0.33 | -0.24 |
|  | CRI | 0.41 | 0.13 |
|  | GEC | 0.47 | -0.05 |
| CBCL | Int | 0.41 | 0.06 |
|  | Ext | 0.10 | 0.38 |
|  | Tot Pro | 0.19 | 0.26 |
|  | Anx/Dep | 0.31 | -0.15 |
|  | With/Dep | 0.35 | -0.11 |
|  | Som Com | 0.22 | 0.22 |
|  | Soc Pro | 0.31 | -0.14 |
|  | Tho Pro | 0.02 | 0.64 |
|  | Att Pro | 0.32 | -0.14 |
|  | RB Beh | 0.19 | 0.25 |
|  | Agg Beh | 0.08 | 0.41 |
|  | Affect Pro | 0.18 | 0.27 |
|  | Anx Pro | 0.10 | -0.39 |
|  | Som Pr | 0.24 | 0.20 |
|  | ADHD | 0.46 | -0.03 |
|  | ODP | 0.23 | 0.22 |
|  | Con Pro | 0.13 | 0.33 |
| TSCYC | Anxie | 0.02 | 0.65 |
|  | Depre | 0.00 | 0.88 |
|  | Anger | 0.15 | 0.29 |
|  | I-PTS | 0.17 | 0.27 |
|  | Av-PTS | 0.16 | 0.28 |
|  | Ar-PTS | 0.03 | 0.58 |
|  | Tot-PTS | 0.02 | 0.59 |
|  | Diso | 0.11 | 0.34 |
|  | Sex | 0.30 | 0.15 |
